# WordCommentsAnalyzer: A windows software tool for qualitative research

**DOI:** 10.12688/f1000research.14819.2

**Published:** 2018-09-04

**Authors:** Ehsan Abdekhodaie, Javad Hatami, Hadi Bahrami Ehsan, Reza Kormi-Nouri

**Affiliations:** 1 Department of Psychology, University of Tehran, Tehran, Iran; 2 Center for Health and Medical Psychology, Örebro University, Örebro, Sweden

**Keywords:** Computer assisted qualitative data analysis software, Microsoft Word, comments, coding, thematic analysis, code hierarchy tree

## Abstract

There is a lack of free software that provides a professional and smooth experience in text editing and markup for qualitative data analysis. Word processing software like Microsoft Word provides a good editing experience, allowing the researcher to effortlessly add comments to text portions. However, organizing the keywords and categories in the comments can become a more difficult task when the amount of data increases. We present *WordCommentsAnalyzer*, a software tool that is written in C# using .NET Framework and OpenXml, which helps a qualitative researcher to organize codes when using Microsoft Word as the primary text markup software. *WordCommentsAnalyzer* provides an effective user interface to count codes, to organize codes in a code hierarchy, and to see various data extracts belonging to each code. It also offers basic visualization tools. We illustrate how to use this software by conducting a preliminary content analysis on Tweets with the #successfulaging hashtag. We also demonstrate that the software has satisfactory performance on a large dataset of Iranian journals abstracts. We hope this open-source software will facilitate qualitative data analysis by researchers who are interested in using Word for this purpose.

## Introduction

Commercial qualitative data analysis (QDA) software tools such as NVivo, MAXQDA and Atlas.ti seem to be the most popular in the qualitative research community ^1, 2^, especially in health research. For example, a study found that 763 published articles in the Scopus database (between 1994 and 2013) used Atlas.ti and NVivo in their work, and that the majority of these studies were published in health sciences journals ^3^. However, learning to use these complex software tools may be inconvenient for some researchers. In fact, research has shown that learners of complex qualitative tools often struggle with confusions, frustrations, and feelings of inadequacy ^4^. Moreover, using complex QDA software may create a feeling for the researcher that they are forced to work within the software structures ^5^. Besides, the purchase of commercial QDA software may not be affordable for some researchers. On the other hand, free or open-source solutions that are available often do not provide a smooth editing and markup experience (e.g., QDA Miner Lite does not support Persian and Arabic languages; CATMA and CAT ^6^ are not fast due to their web-based nature). For these reasons, some researchers use professional word-processing programs for their qualitative research projects.

The use of Microsoft Word for QDA is commonly documented ^7, 8^. Using Word comments provides a straightforward way to annotate specific portions of the text and attach keywords or categories (codes) to them. However, as the amount of data grows, organizing codes in Word comments becomes an exhausting task.

In this article, we present *WordCommentsAnalyzer*, a free, open-source tool that allows qualitative researchers to automate organization of the qualitative codes through a fast and easy-to-learn graphical user interface (GUI) while coding the textual material using Microsoft Word as professional, familiar word-processing software.

## Methods

### Implementation

**Figure 1.**
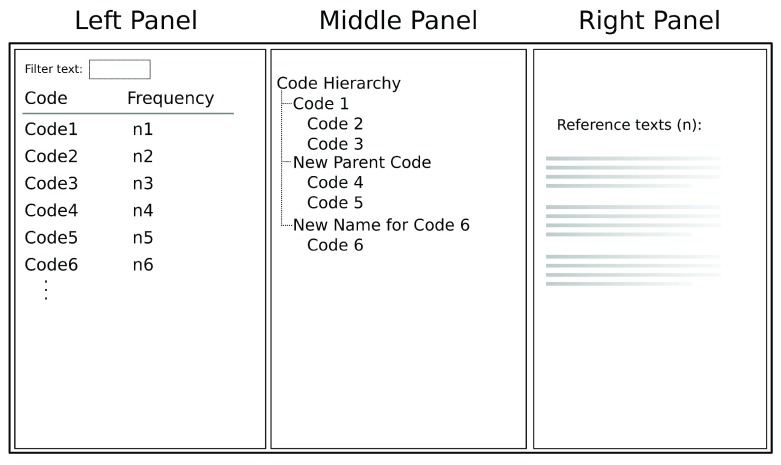
An illustration of the three side-by-side panels of the *WordCommentsAnalyzer* graphical user interface (GUI). The left panel shows the codes in the comments with their counts, the middle panel provides a code tree for intuitive organization of the codes and the right panel shows the data extracts pertaining to each code (or to children of a parent code). The code list in the left panel can be filtered to find specific codes. The user can place codes in the code hierarchy simply by using drag-and-drop. The tree also enables the user to move codes in the hierarchy if needed. The user can introduce a new parent code. The codes are changed or combined by being wrapped in new codes.

This software is written in C# programming language using .NET Framework 4.5.2. The software also makes use of OpenXml library to extract comments from Word documents. Recent versions of Word store documents in XML format. OpenXml provides an easy way to query comments from a document. To facilitate assigning multiple codes to a piece of text, we assume a simple convention: different codes are entered in a comment with line breaks between them (as the descendant paragraphs of the comment element). The software uses a relational model approach to store the extracted codes and uses language integrated queries to collect different text portions related to each code, to calculate the code frequencies and to sort the codes by frequency. The main visual interface of the program consists of three side-by-side panels ( Figure 1). The left panel shows the codes in the comments with their counts, the middle one provides a code tree that the user can intuitively organize their codes in and the right panel shows the data extracts pertaining to each code. In the left panel, the code list can be filtered to find specific codes. The user can place codes in the code hierarchy simply by using drag-and-drop. The tree also allows for moving codes in the hierarchy if needed. The user can introduce a new parent code or a code that is of a higher level of abstraction. Additionally, the codes are changed or combined by being wrapped in new codes. The code hierarchy tree is saved as a tab-indented text file in the data folder (codehierarchy.txt). The tree is auto-saved every minute and can also be manually saved by clicking Save button. The previous tree files are backed up in a subfolder of the data folder. When a collection of codes develops after coding several documents, the user can drag and drop the codes into the word-processing software to avoid memorizing them. In addition to organization tools available in the GUI, the software offers two visualization tools: Code Co-Occurrence Matrix visualizes the number of co-occurrences of sets of two codes in the data and File Code Matrix visualizes the number of occurrences of each code per Word document.

### Operation

The requirements for this software are Windows 7 or later and .NET Framework 4.5.2. After installing the .NET Framework, the user can unzip the latest release package from the GitHub link and run the “WordCommentsAnalyzer.exe” executable file. The program supports XML Word documents (using the .docx extension). Older Word documents (using the .doc extension) can be easily converted to XML documents by Word 2003 or later (there are also resources available on the web to batch-convert older Word documents). The program allows multiple Word files to be analyzed. This feature can be utilized to separate transcripts of different interview or focus group sessions into different files.

## Use case 1

**Figure 2.**
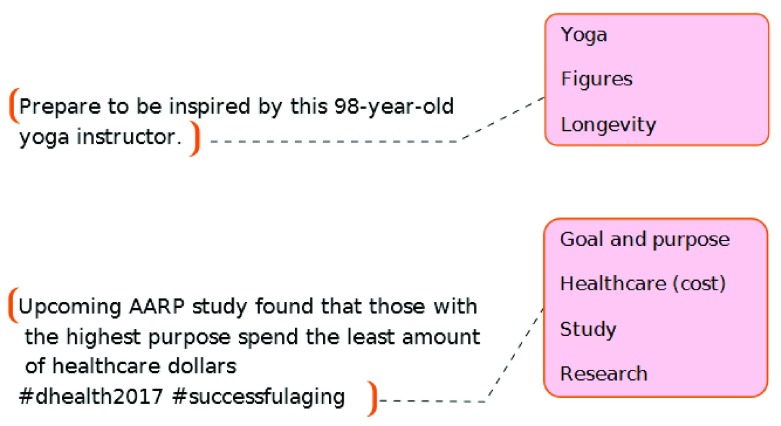
Two text samples of #successfulaging Tweets, which are commented using line-break-separated codes. The codes describe notable topics concerning the text samples.

To illustrate how to use the software, we first present a mini-study of Twitter’s Tweets from 17 January 2017 to 10 April 2018. The Tweets with the #successfulaging hashtag were copied into two Word documents based on the year in which the Tweets were posted ( Supplementary File 1). We reviewed the Tweets and added comments (line-break-separated codes) to portions of texts containing interesting notions related to successful aging. Two examples of these text portions are reproduced in Figure 2.

**Figure 3.**
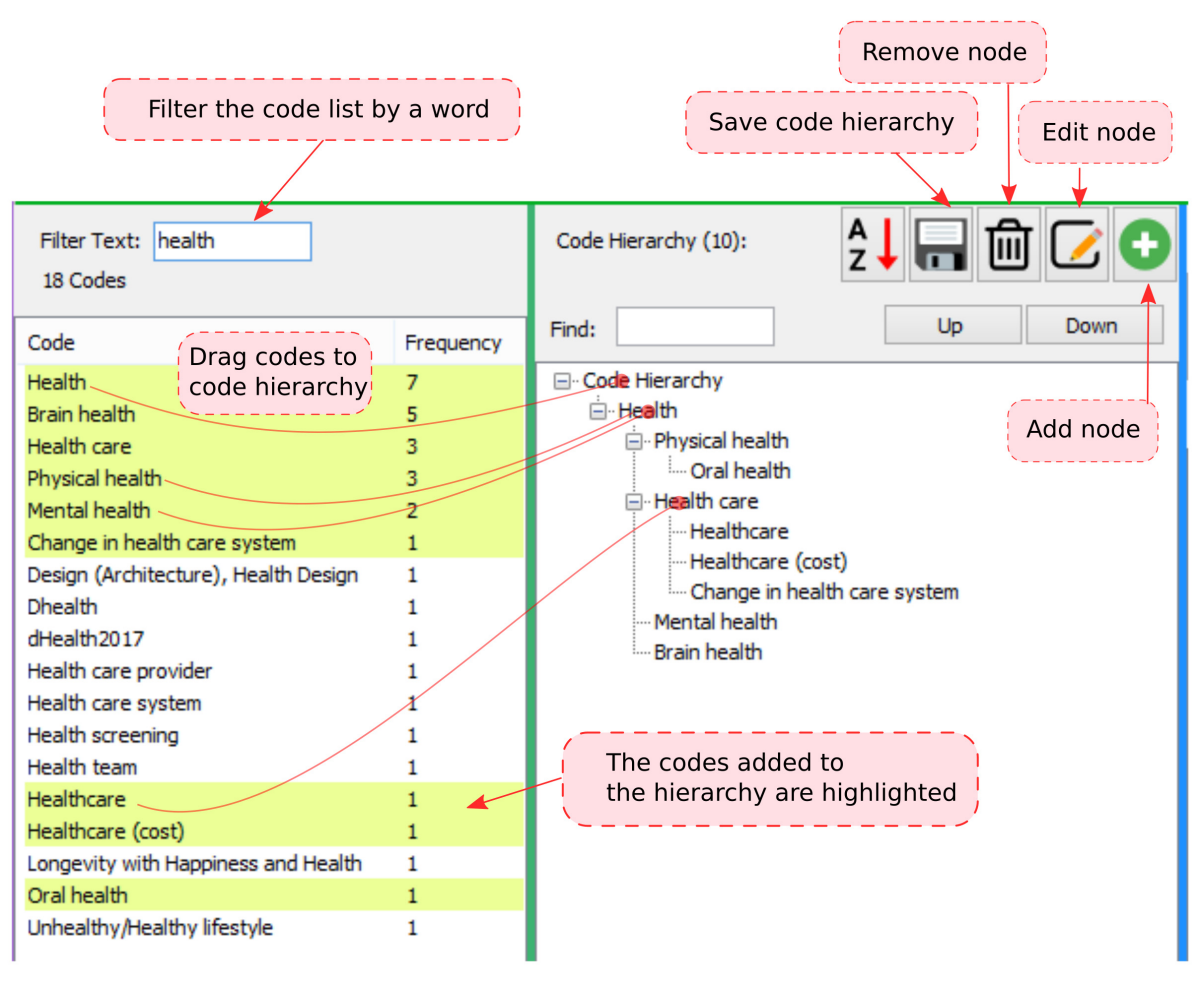
Basic features in the left and middle panels of the *WordCommentsAnalyzer* graphical user interface (GUI). The user can find specific codes by filtering the code list (e.g., by the word of “health”) and organize the codes (from the left panel) by dragging and dropping them into the code hierarchy tree (the right panel).

After adding comments to Word documents, we run *WordCommentsAnalyzer*, select the folder containing the Word documents and click *Analyze*. The program analyzes the comments and shows a list of codes with their counts in the left panel. The middle panel enables us to organize the codes by placing them in a code hierarchy ( Figure 3). For example, we can find several codes related to health by filtering the code list by the word of “health”. Then we add the code of “Health”, which is a parent code, to the hierarchy by dragging and dropping it onto the root node (“Code Hierarchy”) or the empty area. The codes of “Brain health”, “Physical health”, and “Health care” can then be drag-and-dropped onto the node of “Health”. Likewise, “Oral health” is inserted into “Physical health”. When organizing the codes, we could check the right panel to assure the data extracts support the codes. Also, the codes inserted into the hierarchy will be highlighted in the code list to help keep track of the organized codes.

**Figure 4.**
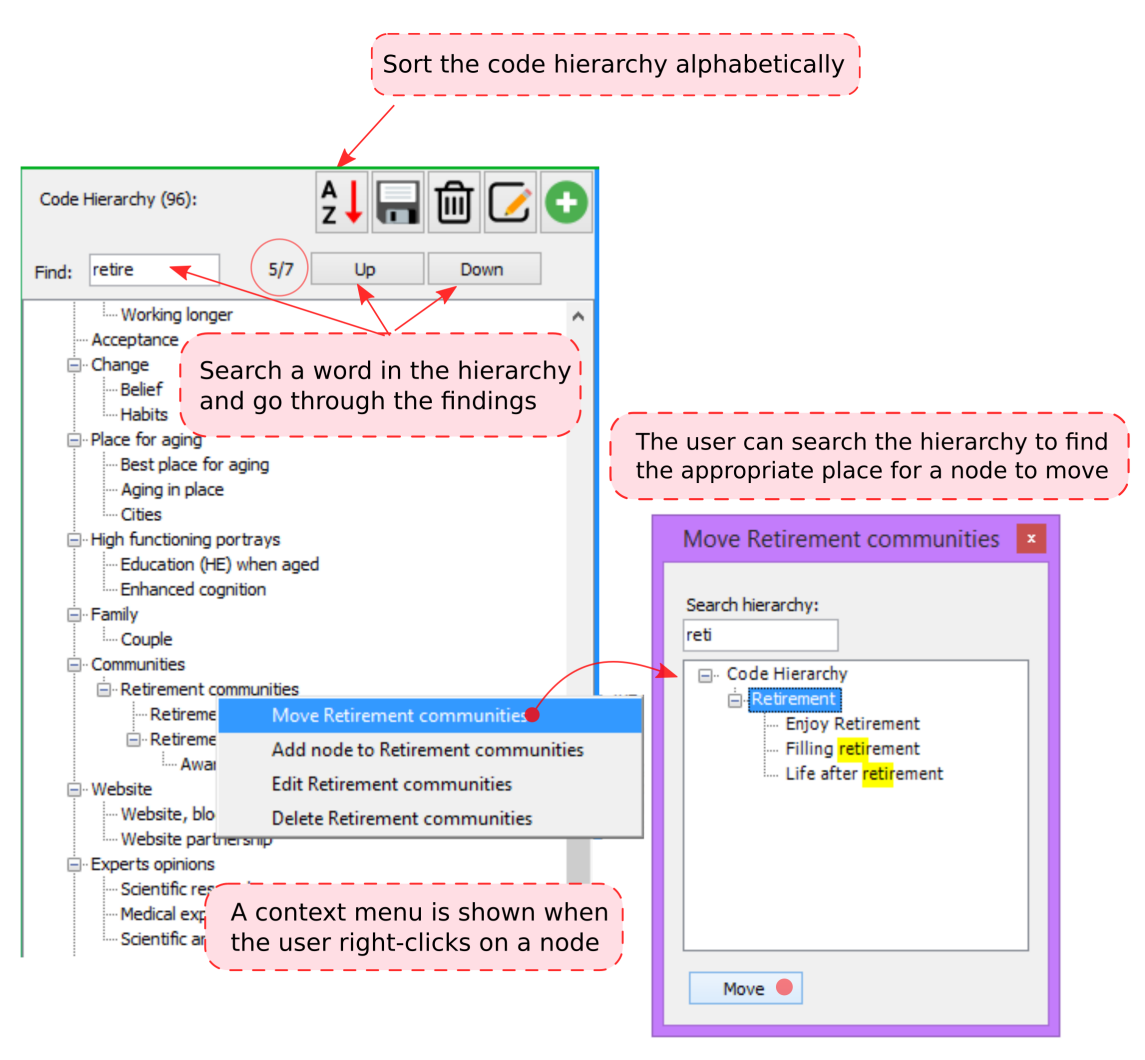
Search and move code features of WordCommentsAnalyzer which facilitates reorganization of codes in the code hierarchy. WordCommentsAnalyzer facilitates finding and moving specific codes by two features: 1) the user can search particular words in the hierarchy; 2) the user can move codes to other codes that are not visible in the current view by means of a pop-up window.

As the number of codes in the code hierarchy increases, moving or reorganizing codes becomes cumbersome, particularly when the user intends to move a code to another distant code or find specific codes in the deeper branches of the hierarchy. The software offers two features for smooth reorganization of codes: search specific words in the hierarchy and move codes through a pop-up window ( Figure 4). Consider we want to review all the codes containing “retirement” in the Tweets data. We type a portion of this word (“retire”); by looping through the results (clicking Down), we realize that the “Retirement communities” is currently a child node of the “Communities”. Thinking that this node better suits the “Retirement” node, we can right-click it and select Move Retirement communities, then search for the “Retirement” node in the pop-up window, and move the “Retirement communities” to its appropriate place.

**Figure 5.**
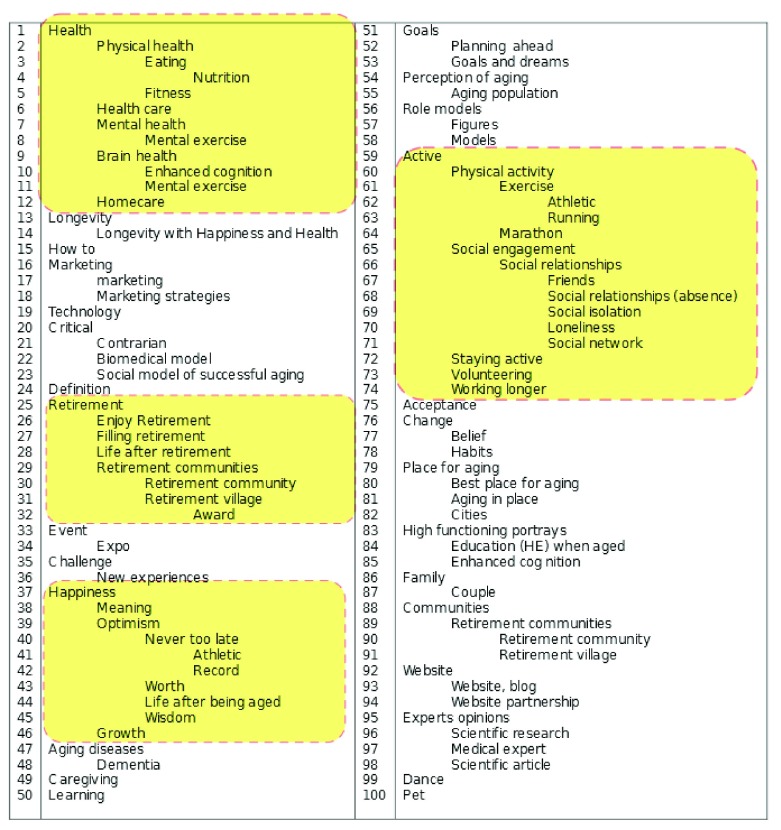
A formatted version of tab-indented text output file of the code hierarchy tree. When we organized the Tweet codes with at least two counts. The large branches of the code tree can help the researcher identify the richest themes in the data. Thus, themes of health, retirement, happiness, and being active are probably the major themes in the Tweets with the hashtag #successfulaging.

Figure 5 presents a formatted version of codehierarchy.txt ( Supplementary File 2) when we organized the Tweet codes with at least two counts. As shown in this figure, the themes of health, retirement, happiness and being active represent the richest themes in the Tweets of #successful aging.

**Figure 6.**
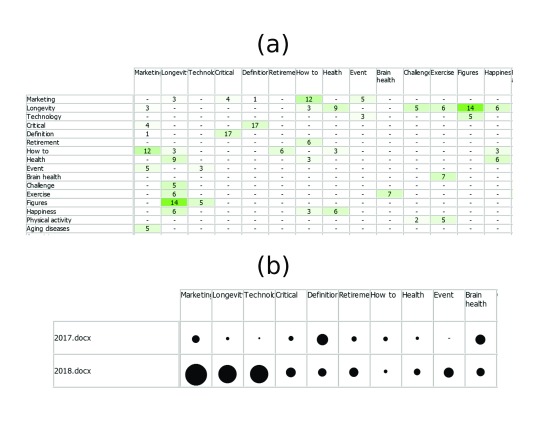
Basic visualization features of WordCommentsAnalyzer: Code Co-Occurrences Matrix ( **a**) and File Code Matrix ( **b**). WordCommentAnalyzer offers two visualization tools. Code Co-Occurrence Matrix enables the researcher to recognize patterns of codes co-occurrence in the data. The numbers in the co-occurrences matrix are counts of text segments that share the corresponding pair of codes and colors of the cells reflect c-coefficients ^9^. High co-occurrence suggests thematic proximity between a pair of codes. For instance, in the co-occurrence matrix generated by the software for the Tweets data ( **a**), there is a high co-occurrence between the “Longevity” and “Figures” codes, which shows that the Tweets tend to present model figures as very aged. File Code Matrix assists the researcher in inspecting the various data parts (e.g., different interview or focus group sessions) in terms of the codes or themes they contain. For example, this figure ( **b**) demonstrates that while the coder(s) coded more Tweets with “Longevity” than they coded with “Brain health” in the 2017 document, this pattern was reversed in the 2018 document.

WordCommentsAnalyzer also allows for getting basic visual representation of the data. By clicking Visualize, a new window with two tabs appears. In Code Co-Occurrences Matrix tab, we see two identical instances of the codes lists. By checking codes in these lists, the software builds a co-occurrence matrix with the checked codes in the lists as the columns and rows. The numbers in the matrix cells show the number of text segments that share the corresponding pair of codes and the cells’ color intensities are associated with the c-coefficients ^9^. Creating the co-occurrence matrix for the Tweets data, allows inferring some thematic proximity between the codes with high co-occurrence. For instance, that the codes of “Longevity” and “Figures” have relatively high co-occurrence shows the Tweets’ tendency to present model figures as very aged (see Figure 6a). Also, the high co-occurrence between “Marketing” and “How to” may indicate that the Tweeters often use “How to” phrases for marketing purposes.

In the File Code Matrix tab, we can generate a matrix of the number of paragraphs with a certain code in each document. For example, the matrix in Figure 6b demonstrates that while the number of Tweets coded with “Longevity” was smaller than ones with “Brain health” in 2017, the former is greater than the latter in 2018.

## Use case 2

The purpose of this use case was twofold: to test the performance of WordCommentsAnalyzer against a large dataset and to test the software when working with Persian/Arabic texts.

We collected the abstracts of eight Iranian journals in health sciences published until Aug 2018. We collected each journal issue into a Word document and assigned the keywords as codes for each abstract (All the journals were licensed by Creative Commons, CC BY 4.0 or CC BY-NC 4.0; the commented Word files and code hierarchy text file are available in Supplementary File 3). The dataset was quite large, comprising 388 files, 4624 paragraphs, and 10378 codes. We tested the software on an ASUS U41J laptop (Intel(R) Core(TM) i5 CPU M480@2.67GHz processor; Hitachi SATA/300, 5400 RPM hard drive). The analysis was completed in a few seconds. We organized 1000 frequent codes into the code hierarchy. Although the number of the nodes was large, all the panels remained responsive and the search functions responded almost instantly. Also, the visualization tools had decent performance; creating The Code Co-Occurrence Matrix took a few seconds when the matrix was smaller than 100x100 and took less than 30 seconds when it was as large as 500x500; the software created File Code Matrix for 1000 codes and 388 files in about 30 seconds. The software also performed well in all the operations including code search when working with Persian/Arabic characters.

## Comparison of WordCommentsAnalyzer with other available tools

As mentioned at the introduction, WordCommentsAnalyzer is based on the idea that users code the textual data in the word-processing software and subsequently organize the open codes in an effective user-friendly environment. Thus the users of this software are not able to do analysis on non-textual data such as images, audio, and video. Besides, the users must do operations of coding, re-coding, and removing codes on the word-processing side. Therefore we compared only analytical capabilities of the software for textual data (i.e., what is done after open coding) with other QDA software ( Table 1). In contrast to other tools, WordCommentsAnalyzer provides no memo-writing features. It offers features to count codes, to do simple queries on the codes and to organize them in a hierarchy. However, it does not allow complex queries (e.g., using Boolean or proximity operators). The recent version of the software generates basic visualizations such as Code Co-Occurrence Matrix and File Code Matrix but does not provide sophisticated visualizations like mind mapping tools. It is noteworthy that, RQDA (another free, open-source software) offers similar features as well but not in the GUI (the user have to write R syntax).

**Table 1.** Analytical capabilities of some well-known QDA software along with the *WordCommentsAnalyzer* capabilities. The table presents analytical capabilities of three popular commercial QDA software tools and one free, open source QDA program along with features of WordCommentsAnalyzer. Because the focus of WordCommentsAnalyzer is on the analytic work after coding data, we did not include features of these software tools used for raw data manipulation and/or coding. Although WordCommentsAnalyzer offers no memo-writing or mind-mapping features, it provides features to do simple queries on the codes and to organize them in a hierarchy. In addition, it provides basic visualization tools.

MAXQDA 2018 (Commercial)	
Create memos and attach to the codes or data	• The user can create free and attached memos (attached to documents, codes, etc.) • The user can log notes about the research process in the logbook. • Other writing tools included are paraphrases, comments, and summaries.
Search and interrogate the dataset	• The user can see frequency of codes across the dataset. • The software provides Boolean and proximity operators for retrieving coded text. • The user can find co-occurrences of codes in up to six dimensions.
Visualize codes and the data	• The user can get a big picture of tones of codes in a document (Document Portrait), see counts of codes per document (Code Matrix Browser) or counts of sets of two codes across the whole dataset (Code Relations Browser). The user can also see how paragraphs in a document are coded (Code Line) and compare this to other documents (Document Comparison Chart). • The software provides MAXMaps which is a tool allowing visual mapping of all aspects of the project. The user can also add items not existent in the project to the map or create a ‘free’ mind map. MAXMaps provides a variety of automatic templates to generate visualizations, e.g., a map of codes hierarchy or a map showing code co-occurrences.
Nvivo 12 (Commercial)	
Create memos and attach to the codes or data	• The user can create memos and link sources to them. • The user can link a source to only one memo.
Search and interrogate the dataset	• The user can see frequency of codes across the dataset. • The software provides various types of queries to search text, find cases with a combination of codes, attributes and/or specific texts • The queries can be saved for later use.
Visualize codes and the data	• A variety of visualization tools including Mind Maps, Concept Maps, Project Maps, Matrix Query, and charts of cluster analyses of word similarity.
Atlas.ti 8 (Commercial)	
Create memos and attach to the codes or data	• The user can create memos and link them to Quotations, codes and other memos. The user can also write Comments which are spaces granted specifically to different project components (e.g., Code Comments).
Search and interrogate the dataset	• The user can search for words in the name, content or comments of the project components (e.g. codes, documents, memos, groups, networks, etc.), with an option to search on the basis of the user that created the components. • The Query Tool provides Boolean and proximity operators for retrieving quotations based on the codes. Additionally, it provides 3 semantic operators (UP, DOWN, SIBLINGS) allowing retrieval based on the presence of transitive links between codes. • The user can retrieve Quotations based on two-code co-occurrences in a tree view (Co-Occurrence Explorer) or a matrix (Co-Occurrence Table).
Visualize codes and the data	• The user can create highly customized networks of the linked components. The user can arrange the components in a network using a number of automatic layouts offered. • The software shows the relationships between codes in the Co-Occurrence Table. The table cells have shades of colors which indicate the strength of the relationship by c-coefficient.
RQDA 0.3-1 (Free, Open source)	
Create memos and attach to the codes or data	• The user can create memos for codes, cases, and files but not for individual data extracts.
Search and interrogate the dataset	• The user can search codes effectively by R commands but cannot search the codes for particular words in the GUI when they code data extracts, organize codes, etc.
Visualize codes and the data	• The software can create a map of codes belonging to code categories and arrange them by a number of automatic layouts.
WordCommentsAnalyzer 2.0.3.0 (Free, Open source)	
Create memos and attach to the codes or data	• The software does not provide the memo-writing feature.
Search and interrogate the dataset	• The user can see frequency of codes across the dataset. • The user can search the code list or code hierarchy for particular words when organizing the codes.
Visualize codes and the data	• The software provides a matrix of code co-occurrences for selected codes. The table cells have shades of colors which indicate the strength of the relationship by c-coefficient. • The software provides the File Code Matrix which visualizes codes in the Word files by a matrix of circles of varying size. The radius of circles inside the table cells represents the relative number of paragraphs coded by different codes in each file.

Note: the authors used software reviews by Silver, Lewins, and Bulloch ^10^ and Silver and Bulloch ^11^ to create some parts of this table.

## Conclusion

The rationale for developing WordCommentsAnalyzer was to facilitate organization and analysis of codes for researchers interested in using Word for data annotation. Despite that the query tools of this QDA software are somewhat limited and it includes no memo-writing/mind-mapping tools, it is free and open-source and provides basic code query and visualization tools through an easy-to-learn GUI. WordCommentsAnalyzer may provide a good option for researchers who see commercial QDA software as too advanced, complex or costly for their research purposes. By using this free software, the qualitative researcher can utilize a convenient word-processing application yet they reduce the efforts of manual organization of the codes.

## Software availability

**Source code available from:** https://github.com/ehsabd/word-comments-analyzer.

**Archived source code at time of publication:** https://doi.org/10.5281/zenodo.1404728 ^12^.

**License:** GNU General Public License 3.0.

## Supplementary material

**Supplementary File 1. Tweets hashtagged with #successfulaging from 17 January 2017 to 10 April 2018.**

**Supplementary File 2. The tab-indented text file of code hierarchy.**

**Supplementary File 3. Coded abstracts of eight Iranian journals in health sciences published until Aug 2018 along with code hierarchy text file.**
