# Supplementary material for: WordCommentsAnalyzer: A windows software tool for qualitative research
Source: F1000Res. 2018 Sep 4;7:536. Originally published 2018 May 3. [Version 2] doi: 10.12688/f1000research.14819.2 (PMC6137411; doi:10.12688/f1000research.14819.2)
Supplement: Supplementary file 1 [file f1000research-7-17614-s0000.tgz › cb3b92b5-0191-4f35-b0ca-81b7a18eabae.zip!/2018.docx]

#PhysicalActivity guidelines for adults - Live Well - NHS Choices - https://www.nhs.uk/Livewell/fitness/Pages/physical-activity-guidelines-for-adults.aspx … #successfulaging

Congratulations, Friendship Village of Bloomington, on becoming a Certified Center for Successful Aging! #successfulaging #MasterpieceLiving https://buff.ly/2CelUIJ

Check out Masterpiece Living President Dr. Roger Landry's podcast interview. He explains what we can do to change the stereotypes of aging in America and how we can transform public policy of ageism. #ageism #aging #successfulaging https://www.spreaker.com/user/birdland_media_works/aging-in-america-the-last-unchallenged-s …

#SuccessfulAging https://twitter.com/schwarzenegger/status/945327325142327296 …

Merry Christmas! I’m grateful for each and every one of you for following my adventures and missions. As a gift, here’s a quick new training montage to inspire you in 2018.

Hillcrest's Grace Knott discusses the importance of exercising both your body and mind. #aging #successfulaging

Memoirs published by Lufkin, TX Pinecrest Retirement Community Writing Club http://www.ktre.com/story/37094451/memoirs-published-by-pinecrest-writing-club#.WjkHh7nrNHo.twitter … #purposeful #successfulaging #Gerontology

She's worked at NASA for 60 years, longer than any other woman ... no plans to retire "unless things start to get really boring." Not likely to happen soon! #successfulaging https://buff.ly/2AqSAAa

Masterpiece Living President, Dr. Roger Landry, shares the benefits of living in a Masterpiece Living partner community. #successfulaging #MasterpieceLivinghttps://buff.ly/2BkQqyI

Give Your Mind a Workout—Try Meditation - The Wall Street Journal #wellness #stressmanagement #successfulaging #mindfulnesshttps://apple.news/AAOXMDEKTSj-TeG-fmIgOzg …

Sue Finley began working at NASA's Jet Propulsion Laboratory in 1958. She has no plans to retire "unless things start to get really boring." We don't anticipate that happening anytime soon! #successfulaging #NASA #spaceexplorationhttps://buff.ly/2AsdkYf

#Resilience - its a skill we need to work on at all phases of life. BHI's Laura Malloy, director of our #SuccessfulAging program, provides some important information on the benefits and building of resilience. http://ow.ly/cqbl30gTL2x

Masterpiece Living and Dr. Roger Landry recently had the opportunity to visit Village on the Green (a Lifespace community) for a very special film project. https://buff.ly/2zSmTQ3  #successfulaging

Presence Horizon Tummy hungry Kid thirsty Storm coming Can you move Baggage Promise Story Blame Ability #successfulaging #aging

US News and World Report recently named Colorado as the Best State for Aging. Boulder, CO was also cited as one of the Happiest Places in the United States. Read more ... #happiness #bluezones #successfulaging https://buff.ly/2zkkNZJ

#TBT Reflect on the transitional process between #Disability and #Aging in #Switzerland at http://bit.ly/RDS_585  #RDSJ #CDS #Disability #Aging #SocialSecurity #SuccessfulAging #DisabilityStudies

Residents and team members at Village on the Green participated in a special film project on behalf of Masterpiece Living. I enjoyed spending time at this great community. #MasterpieceLiving #SuccessfulAging https://buff.ly/2zTyD4E

Thank you to everyone who joined us for the 2017 LeadingAge meeting and Expo! Ready to join the successful aging movement? Click the link below for access to the free "Where You Live Matters" brief. #WYLM #LeadingAge17 #successfulaging https://buff.ly/2A01LEZ

Why we say, “‘#SuccessfulAging is *not* dying. It is *not* hiking Appalachian at 85 w/grandkids & new knees, & if not there also w/RV,& Premium insurance, you have ‘failed at’ #aging, & ‘deserve’ to suffer.”,...(contrary to ‘marketing’, eh?!)https://twitter.com/staceyhankeinc/status/931483382017609729 …

Our culture value and rewards people who are physically fit, appropriately dressed, well groomed and confident.

Bill Gates' newest mission: Curing Alzheimer's @CNN @drsanjaygupta http://cnn.it/2hmvBuR  #SuccessfulAging

#SuccessfulAging #Neoliberalism 'it makes the ageing process appear to be something we can choose to avoid, not something inevitable or desirable'http://www.smh.com.au/lifestyle/news-and-views/all-women-are-disadvantaged-by-the-idea-of-successful-ageing-20171115-gzlsol.html …

#SuccessfulAging This one-day conference features multiple tracks for healthcare professionals & caregivers. Sessions include Alzheimer's related topics, Medicare, polypharmacy, opioid use/abuse, and the 5 Love Languages. Join us on Thursday in Lumberton http://ow.ly/4wy530gzeKA

In SF for @Aging20 #A2OPTIMIZE? Join our Health team for a small reception on innovation for #successfulaging and learn more about #healthus18. More info here:http://bit.ly/2yP8uUU

We're excited to announce a collaboration with @KPCommHealth on #HealthUS18 to support #startups enabling #successfulaging. Learn more here: http://bit.ly/2iInzNp

Leslie Lehnhoff loves learning more as she teaches older adults to play the piano. #music #successfulaging http://bit.ly/2qs7DCl

#SuccessfulAging #Aging cannot be determined by “two new knees & hikes PCT at 90!”, even if more folks can & do do such things as this...https://twitter.com/iramey/status/926390403317690368 …

“Successful Aging” is*not *&*cannot be*some material/class standard set as “The” expectation of worth & value for surviving,& especially now

Laura Malloy, LICSW Director of #Yoga programs, spreading #laughter + #resiliency through #laughteryoga She also directs #successfulaging https://twitter.com/MGH_RI/status/925725248049303552 …

Can laughter yoga help with #stress? Check out this @statnews video featuring @BensonHenry https://www.statnews.com/2017/10/31/laughter-yoga-health-benefits/ … #INSAD2017

Rethinking #SuccessfulAging for National Native American History Month in #jgss https://doi.org/10.1093/geronb/gbw128 … @OUPAcademic @geronsociety

Thank you to @KFNX_Headlines for featuring us on #SuccessfulAging to talk about the #duetsymposium! http://www.successfulaging.info/previous-episodes/ep36 … #AZ #phoenix

Heading to the 2017 LeadingAge Expo? Be sure to visit us at booth #1360! #successfulaging #LeadingAge17 https://buff.ly/2xv3sby

The time is now for implementing senior-specific ED care models #SuccessfulAging @WestHealth @TheIHI https://publichealth.wustl.edu/tailoring-emergency-care-for-older-adults/ …

I had a great time visiting University Village Thousand Oaks. #successfulaging #livelongdieshort

Get Off The Couch Baby Boomers, Or You May Not Be Able To Later https://buff.ly/2wSwLng  #movement #fitness #successfulaging

Meet a remarkable, record-setting 105-year-old cyclist. #successfulaging https://buff.ly/2yd5Ebb

3 changes to Medicare in 2018 that you need to know https://usat.ly/2xL3LhB  @usatoday #caregiving #aging #successfulaging #healthprevention

New paper: #Population-based #longitudinal latent variable analysis of #successfulaging trajectories and #educationhttps://goo.gl/NndSj7

Listening to #StJamesHospital abt @MercerInstitute for #SuccessfulAging. hope there’s a huge emphasis on #nutrition #fitness #stress #sleephttps://twitter.com/atlantic/status/806200289472684032 …

Newly opened Mercer's Institute for Successful Aging follows the philosophy that "#Aging is not just for the old": http://bit.ly/2haCkdk

Instead of Slowing Down, He Revved Up An E-Bike #successfulaging #gerontologyhttps://www.wsj.com/articles/instead-of-slowing-down-he-revved-up-with-an-e-bikeat-70-he-revved-up-with-an-e-bike-1507374019 …

Checkout my latest article on skin & ageing https://www.agelifebalance.com/index.php/2017/10/04/skin_and_ageing/ … #positiveaging #laterlife #successfulaging #seniors #skincare

Scare yourself a litlte every day ... #neuroplasticity #successfulaging https://buff.ly/2xTxaK5

A function-based approach to rehabilitation & therapeutic group work @AOTInews #successfulaging https://twitter.com/cpng_iscp/status/913738810202165248 …

The great work of MISA @stjamesdublin on @RTENationwide tonight 7pm #successfulageing @_ISCP_ #positiveageingweek @AgeAction https://twitter.com/rtenationwide/status/913704774176067584 …

Why You Should Quit Focusing on Hospitality - #successfulaging #retirement #seniorlivinghttps://buff.ly/2jwnQpY

So excited by this report from @NIH #SkyddaCare plans to be right there #successfulaging...listening! @CrystalJonesDMH

@joyfulkira Older Adults’ Perspectives on Successful Aging: https://www.ncbi.nlm.nih.gov/pmc/articles/PMC3593659/ …. Listening to the elderly #successfulaging #agewell

Learn more about how Duet can help you age successfully. http://www.glendalestar.com/features/article_ae207198-94d1-11e7-b88f-f37d49dd1f87.html … #successfulaging #volunteersneeded

Glad to see big crowd yesterday learning how to improve #brainfunction during @JCCManhattan Fall Open House #successfulaging

#successfulaging #gerontology ~~~>https://twitter.com/Tylerpaper/status/907702880085336064 …

CarFit event helps seniors drive safe, learn how to make their vehicles better fit needs. http://bit.ly/2f3Q1Lw

How will you challenge yourself today? #motivation #successfulaging

For Years and Money: 5 Rules for Building True Friendships - https://buff.ly/2wJaNEL  #socialconnection #successfulaging

#Gerontology #Preretirement #successfulaging ~~~> https://twitter.com/TIAA/status/894573866940485632 …

There are 3 things most Americans want in retirement. See in @Forbes what they are and how to get them. https://cards.twitter.com/cards/v9qdu/4mlb7 …

A vision for #SuccessfulAging & how philanthropy can help advance it: @shelleylyford at @WestHealth http://youtu.be/eqEg_vntyUA  @dhealth2018https://twitter.com/dhealth2018/status/903742186725965828 …

Join tech, healthcare & policymakers in Boston, June 6 at the d. health Summit 2018 to promote #successfulaging http://www.dhealthsummit.org/

#aging #successfulaging #babyboomers #GenX #Millennials #innovation #design #ai #brainhealth #market #economy #climate WTF u think happenin?

The latest The Donnamite Pottery Daily! http://paper.li/donnamiers/1495580641?edition_id=eb36ef60-9032-11e7-857d-0cc47a0d1605 … #successfulaging #boomer

An inspirational interview of Lucille Lewin who achieved an MA at age 69 @thatsntmyage #successfulaging #boomer http://bit.ly/2vAO4gN

Join tech, healthcare & policymakers in Boston, June 6 at the d. health Summit 2018 to promote #successfulaging http://www.dhealthsummit.org/

Puppy Power! How can having a pet enhance your successful aging journey! Find out here: https://buff.ly/2x6HjjU  #successfulaging #pets

"I want #socialchange!" "I want #innovation!" "I want #successfulAging!" "I want proven abusers invested in abuse reward to care & do that!" https://twitter.com/iramey/status/902103013031985153 …

"I want to make cinnamon buns!...but I don't want cinnamon, I don't want any recipe, threw away all the flour earlier, destroyed the oven.."

85-year-old Billy Ellis continues fire lookout after 33 years. #successfulaging http://buff.ly/2steJ98

Find out how dogs can support your successful aging journey. #dogs #pets #successfulaging https://buff.ly/2wxKMdn

New report reveals America’s best and worst states to grow old http://bit.ly/2vGEIOU  #successfulaging

A Message from Dr. Roger: Too Old? https://buff.ly/2w6j731  #successfulaging #mindfulmonday

"Whether you think you can or you think you can't - you're right." ~Henry Ford #mindfulmonday #successfulaging https://buff.ly/2vBFMSt

#SuccessfulAging #SuccessfulThesis https://twitter.com/memimachi/status/895838497231167489 …

THESIS DEFENSE DONE!!! #SuccessfulAging @kitkatfernandez @gabnasantwittermo

THESIS DEFENSE DONE!!! #SuccessfulAging @kitkatfernandez @gabnasantwittermo

Count your age by friends #ThrowbackThursday #successfulaging #aging http://bit.ly/1YfeBDP

Jayne Keller on Masterpiece Living and Centers for Successful Aging: https://buff.ly/2waSRCp  @HollyCreek #successfulaging

Compelling! My published #research on #successfulaging highlights the importance of maintaining social networks throughout the #lifespan.https://twitter.com/mnt/status/894289994436706304 …

Loneliness a bigger killer than obesity, say researchers http://bit.ly/2wxJ1to

Find out what it means to live an authentically healthy life. #successfulaging - http://buff.ly/2uQKvSU

Here's how Nora brings out the hidden artist in you! #caregiverstress #alzheimers #successfulaging http://fb.me/3Typky924

Make Your Home a Calorie-Burning Haven - #successfulaging #bluezoneshttp://buff.ly/2utMzNL

Great news- Seems everyone is getting on-board with better ED care for seniors #SuccessfulAging @westhealthhttp://fw.to/GpxR9hB

Do you see the signs? #caregiverstress #familycaregiver #agingwell #successfulaging http://fb.me/uxu5RxIz

Prepare to be inspired by this 98-year-old yoga instructor. #successfulaging #yogahttp://buff.ly/2v7V3ch

We need a new paradigm of #successfulaging #IAGG2017

I was honored to be a part of the Consortium for Older Adult Wellness event. #successfulaging http://buff.ly/2uR93dZ

Experts: One in three cases of dementia preventable https://www.sciencedaily.com/releases/2017/07/170720094907.htm … #Gerontology #Geriatrics #successfulaging

Is humor the secret to #SuccessfulAging? Bob Hope, George Burns,& Betty White are proof that it just might be! http://buff.ly/2uVm4R0

Tune into this stellar interview w/ Dr. Roger Landry on NPR's The Point w/ Mindy Todd.#successfulaging #activelivinghttp://buff.ly/2t3JFxk

How to Get Old Brains To Think Like Young Ones #Gerontology #successfulaging #Lifespandvelopmenthttps://apple.news/Az2i4BlQhRcq0fGxn4Uly8A …

Its #SocialWellnessMonth & research shows our need for friends is essential to well-being & #SuccessfulAging. http://ow.ly/YNJa30dswTt

“I love showing them that there is life after 70,” says Abbey Delray South resident Roberta Hyman. #SuccessfulAging http://ow.ly/7kqB30cTPES

Thank you for sharing, @stanusayale! #successfulaging https://twitter.com/stanusayale/status/881222896907153408 …

I have shared your book with many of my patients!! Live long, live well!

Proper #reflection can lead to reinvention. How will you reinvent yourself? #successfulaging http://ow.ly/rLRx30cQzp8

That's it. - #successfulaging #lifelonglearning #neverstoplearning #jungbleiben #erfolgreichaltern #learningneverstops #geriatricnurse #eld…

Aging Successfully: An interview with Dr. Roger Landry on The Point w/ Mindy Todd. @WCAI_NPR #successfulaging http://buff.ly/2s5xe6P

Via @baptiststandard #Gerontology #SuccessfulAging~~~~~> https://twitter.com/BaptistStandard/status/880393179740098560 …

Older people who feel close to God more likely to have sense of well-being http://bit.ly/2s4Rv9T

Ways to ease the cost of your prescription medication ahead on @myfox8 at 9:00. #SuccessfulAging @retiringsmart #getsmarttriad #upwithFOX8

Save the date for the COAW annual fundraiser on July 20. Details below... #successfulaging #livelongdieshort http://buff.ly/2sGkmm2

Tips for a Long, Healthy Life from ‘Longevity Village’ - The Wall Street Journal #gerontology #successfulaging https://apple.news/AkbYIGRjhTNenQSKQ_7O7bQ …

Communication key in getting seniors to accept nursing care at home @Robert_rosati @kathybowlesrn #SuccessfulAging http://wapo.st/2sHWmRR?tid=ss_tw-bottom&utm_term=.f757fadd07c0 …

GeronMedia: Is #SuccessfulAging different for African Americans with #T2D? Find out here: https://doi.org/10.1093/geronb/gbw119 … #JUNETEENTH2017 geronsoc…

Why aim for #HealthyAging or #SuccessfulAging when you can do #EpicAging! (found at Yoga Barn, Bali) @WallaceLindsay @olga_theou @krockdoc

For successful ageing, control breathing stress, exercise stress and digestive stress #successfulageing #successfulaging

At 85-years-old, Bill Ellis isn't planning to slow down anytime soon. #successfulaging #fire #forestshttp://buff.ly/2qKD892

"The spiritual eyesight improves as the physical eyesight declines." -Plato #SuccessfulAging

#SuccessfulAging starts now @westhealth. These unhealthy habits could destroy your retirementhttp://cnb.cx/2rZTeRl

#aging #ageing #Successfulaging There IS NO WAR BETWEEN GENERATIONS,... & bloody well do not keep trying to make one. #efficiencyhttps://twitter.com/iramey/status/872443903001083904 …

(Or, more to the point,when/if *challenges*/'hard times', why appeal to false hopes false & cheating authorities?! Unless, 'prisoner' to'em) https://twitter.com/iramey/status/872443395427385345 …

Seniors are wonderful #rolemodels and we don't take them for granted at Lifespace. #SuccessfulAging #DisruptAging http://ow.ly/C1G830cgpZy

Start thinking about care episode as a journey not a project says @MargheritaLabson #homehealth for #successfulAging http://homehealthcarenews.com/2017/05/how-home-health-is-getting-on-board-with-the-value-based-market/#.WTGvJ5TDG3E.twitter …

#VitalAging2017 who's going? We'll be at the Escondido location! Free event-plz share! #aging #agewisely #successfulaging

5 things you need to know about the global pension crisis http://buff.ly/2scNsc0  #PensionCrisis #SuccessfulAging #PushLiving

How can we make this easier on families? #SuccessfulAging @westhealth http://www.huffingtonpost.com/entry/7-lessons-learned-in-the-world-of-eldercare_us_59288083e4b07d848fdc038a?ncid=engmodushpmg00000004 … via @TheScope

@getold profiles these three women who #DisruptAging by making the world a better place. #SuccessfulAging http://ow.ly/Df6V30c4q3r

Health care villages and districts create caring communities http://buff.ly/2rzHN2A  #HealthyAging #SuccessfulAging #CareCommunity

#IntergenerationalPrograms are on the rise, and Lifespace already has an edge on that. #SuccessfulAging http://ow.ly/IXWO30c0Suu

Congratulations to this year's Valuing Gray video contest winners! #valuinggray #masterpieceliving #successfulaging http://buff.ly/2ql6Xg9

What's #successfulaging? Here's a good place to start. @SUNYBuffaloLaw @UBuffalo @UBCommunity #IPE #transdisciplinarity #interdisciplinarityhttps://twitter.com/Rock_Health/status/862458043669258241 …

A visual of the roadmap to #successfulaging according to @WestHealth at #dhealth17. What role does technology play?

.@UBuffalo started a big, inspiring conversation on #successfulaging this week ... watch out world! @SUNYBuffaloLaw @UBCommunity #HereIsHow

6 Things You Must Know About Aging in Place http://buff.ly/2rg1vQJ  #AgingInPlace #SuccessfulAging

If affordability is a major factor in your relocation search consider moving to Columbia, MO - #SuccessfulAging http://bit.ly/2rInahq

Our members attended @SunSentinel #SuccessfulAging Expo 5/13 & learned #Club55 is the biggest kept secret! http://ow.ly/svmm30bOq1M

@LashTimothy If we don’t address #oralhealth for seniors, we cannot achieve #successfulaging in this country

Designing for #MemoryCare, senior-living facilities http://buff.ly/2qQqNVy  #Dementia #HealthDesign #SeniorLiving #SuccessfulAging

Highlights from important #aging event @dhealth2017 Summit #SuccessfulAging #dhealth2017 http://www.healthcarenowradio.com/d-health-summit-2017-2/ …

#successday #SuccessfulAging

Here at the #SuccessfulAging Expo in Fort Lauderdale sharing the good news about age reversal and super-longevity!

Challenge yourself and your limitations, and continue to grow regardless of your age. #SuccessfulAging... http://fb.me/5Q4m0CJmK

5 Tips for Enjoying Retirement and Good Living http://buff.ly/2q5FFxv  #RetirementLiving #SuccessfulAging

How Will You Fill 8,000 days of #Retirement? http://buff.ly/2r2gDx2  #SuccessfulAging #HealthyAging #ThePlatinumYears

@Rock_Health: 'A visual of the roadmap to #successfulaging according to @WestHe…

A visual of the roadmap to #successfulaging according to @WestHealth at #dhealth17. What role does technology play?

Starting now at @dhealth2017 summit: @SaraEshelman1 joins panel on "Investing in the future of healthcare". #SuccessfulAging

And BOOM goes the dynamite... "Design WITH me, not FOR me" - June Fisher, @Aging20 Chief Elder Officer #SuccessfulAging #dhealth2017

rt @Underpaidnurse #PatientExperience #AlzheimersDisease #SuccessfulAging #dhealth2017https://twitter.com/Underpaidnurse/status/862360361726394368 …

Thanks @RN_Solutions! will definitely check it out. Finalizing our MVP and looking for pilot sites now. #Alzheimers #ALFS https://twitter.com/startup_chick/status/860944030082908160 …

@LashTimothy Tremendous opportunity to reorganize and redeploy resources to enable #successfulaging vs. creating new #dhealth2017

Upcoming AARP study found that those with the highest purpose spend the least amount of healthcare dollars #dhealth2017 #successfulaging

"Lifecare, not just healthcare. Design with, not just for." @WestHealth #dhealth2017 @dhealth2017 #successfulaging

@LashTimothy We have to think and act differently if we're going to achieve #successfulaging by 2030 #dhealth2017

Love the great work we're doing for seniors @westhealth #SuccessfulAging https://www.youtube.com/watch?v=dgqaXvTxH68&feature=youtu.be&t=1m10s …

#pinksocks #GSD #dhealth2017 #SuccessfulAging

Health innovations can - and are - creating better patient outcomes! Learn more at @dhealth2017 summit! #SuccessfulAging

Only 4 seats left! Can't make it? Stream the http://d.health  Summit LIVE! https://goo.gl/6x34Df  #SuccessfulAging #dhealth2017

POLL: How would low-income Aging Americans fare under the proposed health care act? #dhealth2017 #SuccessfulAging

2 days until @dhealth2017 summit! @SaraEshelman1 will be in NYC to talk #ImpInv, #HealthTech & more http://www.dhealthsummit.org/agenda/  #SuccessfulAging

Leslie Lehnhoff learns more every time she gives older adults piano lessons. #music #successfulaging http://ow.ly/9NJX30bq0hg

Love love this video! #successfulaging #marriedforseventyyears http://fb.me/23pHhqDaZ

Attending @dhealth2017 summit? Join @SaraEshelman1 & others for discussion on Future of Healthcare http://www.dhealthsummit.org/agenda/  #SuccessfulAging

Join us on May 13th for a a full day of expert advice, health screenings, and more! Details at http://provhealth.org/601485CG0  #successfulaging

Learning to Sing Again http://buff.ly/2oWjhYy  #lamellarIchthyosis #SuccessfulAging

At 71 Doreen Pechey became the oldest person to pass Britain's grade six ballet exam! #successfulaging #dance http://ow.ly/R0dp30bbM21

HOW TO BUILD STRONG BONES http://buff.ly/2qnQDj0  #HealthyAging #SuccessfulAging #Exercise

#walk 4 #brainhealth? #Exercise #SuccessfulAging B ur own #SuperHero #National SuperHeroDay @cbsnews http://www.cbsnews.com/news/walk-your-way-to-better-brain-health … via @cbsnews

Ann Nixon Cooper was just cited it her study. At 107 she cast her vote for the 1st AA President. #Resilient #SuccessfulAging https://twitter.com/hoop4you/status/823016327711490048 …

As one of Ann Nixon Cooper's grandchildren, I want to say thanks. And -- my sister marched in Memphis today for my grandmother.

Becoming Your Best Self As You Age - #successfulaging http://buff.ly/2ox1QO4

Recent studies show that running could add years to your life. #health #exercise #successfulaging http://ow.ly/5TnT30aWV6y

New Boomerang Generation: Moving Near an Elderly Parent http://buff.ly/2pVldQU  #HealthyAging #SuccessfulAging #PushLiving

Wanna learn how they eat in the Blue Zones? Click on this issue of the Mosaic for inspiration! #MasterpieceLiving #successfulaging

What does it mean to "get more colorful with age?" Find out here: #successfulaging @livlongdieshort http://buff.ly/2nBjUBX

Strategically planning for your future can help you live a happier and more productive life. #successfulaging http://ow.ly/Gfp230aWNr5

The man behind Big Bird shares what keeps him excited: always having something to look forward to. #successfulaging http://ow.ly/GBUN30aWPWl

Looking forward to the #SuccessfulAgingExpo this Saturday to share #CalMediConnect info! #SuccessfulAging #PurposefulAging #AgingRedefined

10 Reasons to Free Your Inner Artist - Why do arts matter as we age? http://buff.ly/2pMlUba  #SuccessfulAging #PushLife #PushLiving

You heard your grandmother say, "Use it or lose it!" It's a good motto to live by! #successfulaging http://buff.ly/2pjHHYs

50 years after being the first woman to run the #BostonMarathon, Kathrine Switzer does it again! #successfulaging http://ow.ly/TGe430aUU57

Hear from leaders about tech-enabled solutions for #SuccessfulAging @dhealth2017! Use code ROCK20 to save 20%: http://bit.ly/2pqlXND

Working on solutions for #SuccessfulAging @WestHealth to reduce the worry https://youtu.be/DI00a9u7zQg  via @YouTube

5 tips to stay healthy at home http://buff.ly/2op1eZI  #HealthyAging #SuccessfulAging @PressalitCare

Data Revelation: The Masterpiece Living Network is “Upwardly Mobile!” #mobility #balance #successfulaging http://buff.ly/2oJzG1N

What Would You Do If Your Future Held Infinite Growth Potential? https://backfortyfliers.com/2017/04/08/what-would-you-do-if-your-future-held-infinite-growth-potential/ … @backfortyfliers #gerontology #successfulaging #ngo

The Milken Institute's Best Big And Small Cities For Successful Aging http://ow.ly/Psbc30aGjAY  via @Forbes #SuccessfulAging #AgingInPlace

Ann Trowbridge is a resident of Mirador. In this video, she shares her love of golf. #successfulaging http://buff.ly/2nbFRY5

Tune into my interview on "Which Way is Life" and discover key components to healthy aging. #successfulaging http://buff.ly/2mjq7o3

This Doctor Broke the Law to Build a Better Nursing Home - Let's Rethink Aging!! http://buff.ly/2p7VjVU  #PushLife #SuccessfulAging

#SuccessfulAging is an ongoing process of rejuvenation. Thanks to WhiteHole for this inspiration. http://fb.me/64fuzY07V

Thank you McKnight's Senior Living for mentioning "80 Things to Do When You Turn 80." http://buff.ly/2nZIgYY  @_McKnightsSL #successfulaging

.@GIAging members on all-star @dhealth2017 Summit roster Register http://bit.ly/2mtuv5n  Disruptive tech-enabled solutions #SuccessfulAging

We are looking for volunteers! Call us if you want to make a difference. #volunteering #weloveourvolunteers #successfulaging

10 Challenges from the Environments for Aging Conference: #successfulaging #stress #purposehttp://buff.ly/2neucrB

Check out the DayBreak door! #activeandhealthyageing #successfulaging #fun

We're thrilled to introduce u to our new website! http://www.westhealth.org  is updated to reflect our focus on #successfulaging, check it out!

For #SuccessfulAging u need a solid social network! >> Success is Creating Your Community http://ow.ly/NyBp30akvFq  via@RebrandingAging

The weather is getting warmer! Come and try out our Senior Fitness Park and bring your friends! #successfulaging #fitnessatanyage

Positive role models reduce negative views on aging #SuccessfulAging @unil @FordhamNYC @GeorgiaStateU @geronsociety http://bit.ly/2nIbBYa

Look forward to a happy retirement by planning ahead! #retirement #babyboomer #financialplanning #successfulaging https://twitter.com/AgeLite365/status/846049863997538304 …

#Retirement Tips & Strategies for Working #Women http://fxn.ws/2o0raYV  #Workingmums #financialplanning

self-fulfilling prophesy: having goals and dreams in #OldAge promotes #HealthyAging & #SuccessfulAging, a measure of which is goals & dreams https://twitter.com/AgeLite365/status/846040299340161024 …

"You are never too old to set another goal or to dream a new dream..." ~ C S Lewis #Quotes #ThinkBIGSundayWithMarsha …

The Best Big and Small Cities for #SuccessfulAging http://ow.ly/6Gyy30a1jyv  @NextAvenue

Challenge yourself and your limitations, and continue to grow regardless of your age. #SuccessfulAging... http://fb.me/6ic6pWasx

Come join us at the Home and Leisure Show and learn about our complete selection of aging services! #activeaging #successfulaging

MT @DrKristieLeong #Exercising linked w longer telomeres & less #lossofbrainvolume, both markers of #successfulaging

#SuccessfulAging move from success to significance

A must-watch video! A Masterclass in Active Longevity - #nevertoolate #successfulaging http://buff.ly/2nIH9O2

Thank you! #discount #aging #dhealth #SuccessfulAging https://twitter.com/WestHealth/status/844642458793263104 …

Next important #aging event! Use WestHealth promo code to save 25% #dhealth2017 https://twitter.com/GIAging/status/844640935719919621 …

GIA members on all-star @dhealth2017 Summit roster Register: http://bit.ly/2mtuv5n  Disruptive, tech-enabled solutions for #SuccessfulAging

The older we get, the more optimistic we are about the aging experience. @WestHealth @NORCNews @ASAging #AIA17 #successfulaging

When it comes to #successfulaging, it is more than just medical care that is important. @WestHealth @NORCNews @ASAging #AIA17

More Americans believe signs of old age are factors related to losing to independence @WestHealth @NORCNews @ASAging #AIA17 #successfulaging

Carol Raphael from @WestHealth nat'l survey release-The fatest growing cohort of facebook users is 55 and over #AiA17 #successfulaging

Never say you can't. #GRIT #SuccessfulAging https://twitter.com/AGPTtweets/status/844373830265196544 …

Passion and perseverance two key components of success. http://fb.me/8NYj2h5Zj

Some ways to take care of your brain as you age https://on.dana.org/2nh2bU1  #successfulaging #stayingsharp

Learn more about how our pets can keep us physically and emotionally healthy. #pets #successfulaging http://buff.ly/2mQHsDp

What does #successfulaging mean across the decades? Join our free 3/22 webcast for national survey release at #AiA17http://westhealthagingsurvey.eventbrite.com

Join world-class speakers from tech/#healthcare/policy @dhealth2017 #SuccessfulAging Register: https://goo.gl/QZZVos

Abbey Delray South partners with FAU to mentor students and teach them team-based care. #successfulaging http://ow.ly/aXZ0309TnFl

They ay kids laugh 5 times more than adults. At Green Hills there is lots of laughter in both groups. #successfulaging #laughingthroughlife

Look on the bright side and maybe even live longer http://buff.ly/2mhx0DP  #optimism #aging #healthyaging #successfulaging #pushliving

Roberta Hyman, an Abbey Delray South resident, is helping future health care providers understand #successfulaging. http://ow.ly/Sc5c309OqkB

Aging hasn't stopped this artist from doing what he loves — he still paints every day! #successfulaging http://ow.ly/gTWg309DFRm

Staying Active is key for #SuccessfulAging http://ow.ly/OjGn309FFI0

Join leaders in tech, policy & healthcare to enable #SuccessfulAging @dhealth2017. Seats are limited – register now! http://www.dhealthsummit.org/register/

24-hr FLASH SALE - $100 off registration w/ promo code FLASH100 https://goo.gl/i1kiLS  Hurry - rates go up in 2 days! #SuccessfulAging

Tune into "Which Way is Life" to listen to a recent interview with MPL President, Dr. Roger Landry #successfulaging http://buff.ly/2mj0X9e

The #TechyTeens-SavvySeniors shared high school memories today. #leadingageohio #WellBeyond60 #successfulaging

Early bird pricing ends this week! Join leaders in tech, policy & healthcare to enable #SuccessfulAging @dhealth2017 http://bit.ly/2lF6WRO

Read more about older adults who defy aging stereotypes. What dreams are you ready to fulfill? #successfulaging http://buff.ly/2mBmhVu

It may not be easy, but it's never too late to learn something new! #successfulaging http://ow.ly/UpdF309DEjV

Centenaris, només cal mirar els titulars: vida activa i no rendir-se, entre les claus! #successfulaging

Excited to continue the conversation w/ @Surya_Kolluri 5/10 @dhealth2017 #SuccessfulAging http://video.foxbusiness.com/v/5324000831001/ …

Don’t Let Hazards in Their Home Prevent Aging in Place by Seniors http://buff.ly/2lARKFB  #AgeingInPlace #SuccessfulAging

94-year-old Kung Fu expert, Zhang Hexian, is defying aging stereotypes! #successfulaging #kungfuhttp://buff.ly/2m4i7aD

Pioneering retirement village adds years to residents' lives http://buff.ly/2mbKXG7  #SuccessfulAging #PushLiving

Want to live a long, happy and healthy life? Check out these 10 simple tips for #SuccessfulAging... http://ow.ly/zd99309uijd

Palliative care "raises all ships" says Martha Twaddle at #hdgsummit #successfulAging @WestHealth

*24-hr FLASH SALE* Get $100 off registration with promo code: FLASH100 http://eepurl.com/cD_KXP  #dhealth2017 #SuccessfulAging

Kicking things off at #HDGsummit Looking forward to hearing about enabling tech for #successfulAging

Age Without Borders is in full swing. Click the link to tune in: http://buff.ly/2lhIPIP  #retirement #successfulaging

Did you know that you have a choice about how you age? #successfulaging http://buff.ly/2lgJTgG

#SuccessfulAging 2.0 featured in the latest issue of #JGSS @geronsociety @OUPAcademic https://academic.oup.com/psychsocgerontology/issue/72/2 …

Convening stakeholders and visionaries to look ahead in plans for #aging in #OrangeCounty. #OCSPA #successfulaging

Remeber our blog "The World Is Getting Older"? If not - have look here http://buff.ly/2lu6NCh  #Aging #SuccessfulAging #CareGivers

Staying actively engaged in life is a part of #SuccessfulAging http://ow.ly/68Hu309e97h

Beautiful! Curious Louis: Who is the oldest person in St. Louis? #successfulaging http://www.tinyurl.com/z84kyhj

Do any of you see why there's the very real danger to *anything* #aging #wellness #successfulaging #ageing being handled as any "market"?!

How does our mental and physical health play a role in #SuccessfulAging Flushing Hospital's #DrIraFrankel explains. http://ow.ly/ZPkI3096Yse

#HeartHealth #SuccessfulAging #LivingIt #BlueZones Following the Mediterranean diet provides many heart health...http://fb.me/8MuifV1BF

Older adults around the world are stepping up to create age-friendly cities and towns. #successfulaging http://buff.ly/2jHzMFk

Missed the webinar? Check it out on YouTube: Navigating the Journey of Life #SuccessfulAging http://ow.ly/IEz13092q74

Thank you, @doctorleslie for the encore show! #successfulaging #livinglongdieshorthttps://twitter.com/doctorleslie/status/830069240183021569 …

The Dr Leslie Show http://bridgeholistichealing.com/radio.aspx  Live Long, Die Short!! (Encore)…

It's never too late to learn something new! #nevertoolate #successfulaging http://buff.ly/2kBxqI1

Working everyday @WestHealth to make #SuccessfulAging happen http://sdchamber.org/2017/02/business-leader-spotlight-shelley-lyford/ …

Women getting an education. #SuccessfulAging #Intergenerational relations. Pink saris. These are a few of my favorite things.https://twitter.com/ajplus/status/830768677272244224 …

Grandmas who didn't have access to education growing up are now learning to read and write, thanks to this school.

Thanks for sharing @IlseWiechers Good example of #SuccessfulAging and #SuperAginghttps://twitter.com/IlseWiechers/status/830502065101213697 …

#inspiration #runtastic #RunAustin #ageisjustanumber http://abcnews.go.com/Sports/wireStory/york-man-completed-744-marathons-dies-age-96-45399929 …

We're looking forward to the screening of @liveswelllived at this year's Masterpiece Living Lyceum! #successfulaging https://twitter.com/liveswelllived/status/625499301771214848 …

Official trailer for our documentary film celebrating the secrets, wit, and wisdom of age. https://vimeo.com/132338363  #LivesWellLived

Thrill-Seeking 90-Year-Old Skydives on His Birthday - #neveractyourage #successfulaging http://buff.ly/2jBU0fq

Very excited about joining http://d.health  2017 to enable #SuccessfulAging @dhealth2017 http://ln.is/dhealthsummit.org/iY6af … @health_xl

Better with #age — Robert Marchand has stayed active and at 105-years-old is continuing to excel. #successfulaging http://ow.ly/aor2308QmYc

The Music of Our Lives: http://buff.ly/2ksVRWN  #successfulaging @TheBirches

From @KHNews @susanjaffe: Judge accepts Medicare plan to clear up confusion on therapy coverage: https://goo.gl/GWsjYq  #successfulaging

As world grays, seniors step up to create an 'age-friendly' future: http://buff.ly/2l1bg23  #successfulaging #humancapital

We're looking forward to hearing @livlongdieshort / Dr. Roger Landry on the show! #successfulaging https://twitter.com/WhichWayIsLife/status/827337097660993539 …

Never miss a show! Subscribe to @WhichWayIsLife on iTunes here >> http://apple.co/2eXyyAb

Did you know that you have a choice about how you age? #firesideconversations #successfulaging #mindfulmonday http://buff.ly/2lh19SG

The latest #Flamenco Hoy! http://paper.li/dancehq/1314062062?edition_id=2b3a7d00-ea77-11e6-ba17-0cc47a0d1609 … #successfulaging

"I teach a soft, graceful form of flamenco dancing." A Signal Hill story. #dance #successfulaging #LeadThroughStory http://bit.ly/1LUVbSA

NYU's plan to make college cheaper: get students to live with senior citizens http://buff.ly/2k8g4Ry  #SuccessfulAging #InclusiveSociety

The connection between optimism and #successfulaging is undeniable and backed by numerous studies. http://ow.ly/Zmi1308d3yd

From @MedCityNews: @TrinityHealth expands @VivifyHealth remote monitoring services in 7 states: https://goo.gl/kspNwD  #successfulaging

Government announces interesting #strategy re older workers and helping people retrain #successfulaging #lonliness https://www.gov.uk/government/publications/2010-to-2015-government-policy-older-people/2010-to-2015-government-policy-older-people …

5 Ways Tech Products Will Help Us Age Well: http://buff.ly/2kHUXUw  #technology #successfulaging

#ServingSeniors CEO @PaulDowney22 joins @KPBS @GMWFoundation and #volunteer Fred Davis to discuss #successfulaging

Happy 90th Birthday, Ivan Briggs! Judging from his excitement, I'd say the jump was a huge success. #successfulaging http://buff.ly/2k5ELym

We are all responsible for proper social care http://buff.ly/2jA2oMs  #SocialCare #PushLiving #SuccessfulAging

#ServingSeniors volunteer Fred Davis featured in @KPBS docu series re'flect on #successfulaging http://video.kpbs.org/video/2365926105/ …

10 Best Cities for Successful Aging – Pg1 | https://goo.gl/cYiqhj  | #SuccessfulAging

Masterpiece Living Designates Seven New Certified Centers for Successful Aging. #successfulaging http://buff.ly/2iamfFl

#SuccessfulAging #MeaningandPurpose Our residents were SO happy to be back volunteering at Montessori today. They... http://fb.me/6arT0mUPY

Congratulations! #SuccessfulAging
