# Supplementary material for: WordCommentsAnalyzer: A windows software tool for qualitative research
Source: F1000Res. 2018 Sep 4;7:536. Originally published 2018 May 3. [Version 2] doi: 10.12688/f1000research.14819.2 (PMC6137411; doi:10.12688/f1000research.14819.2)
Supplement: Supplementary file 1 [file f1000research-7-17614-s0000.tgz › cb3b92b5-0191-4f35-b0ca-81b7a18eabae.zip!/2017.docx]

“And in the end, it’s not the years in your life that count. It’s the life in your years.” ~ Abraham Lincoln Have a wonderful Saturday! #SuccessfulAging #OurFamilyTakingCareOfYourFamily

Progressing your crawl pattern is about challenge and fun, children’s playground equipment often provides this. #successfulaging #successfullaging #fitafter40 #fitafterforty #onlinecoaching

"Aging is a precious part of life and we need to do everything possible to engage with it, embrace it and use it as a positive force." #successfulaging https://buff.ly/2FKI5rt

Being willing to accept your new reality, moving forward and aiming to have the best life you can at where you are right now is the secret to happiness. Here are some more tips for joyful living: #successfulaging

Talk about #successfulaging. Former President Jimmy Carter approaches his 94th birthday, and his wife, her 91st.

What role will tech play to enable #SuccessfulAging? https://www.youtube.com/watch?v=ChgIvTbphmg … The human side of #DigitalHealth: Learn How http://Care.Coach  CareAvatarAn Animated Surveillance,VitalPart of the #CareTeam 2Support #Caregivers& #AgingParents #AgeInPlace http://news.trust.org/item/20171023113851-u2etw/ …https://twitter.com/ThistlecreekHC/status/983448369229127680 …

Seven Reasons Why Seniors Want to Stay Home Seven Reasons Why Seniors Want to Stay Home I think that, as http://rviv.ly/4TZuH  #homecare

Did you know that volunteers experience better overall health, more energy and greater life satisfaction? National Volunteer Week starts April 15 - the perfect time to jump in and lend a hand. #volunteer #successfulaging #happiness https://buff.ly/2qjjkvn

Congratulations, Cathedral Village! #bestofbest #masterpieceliving #successfulaging https://buff.ly/2FOJGQt

Sangat sedikit LGBT dengan usia 60 th ke atas yang memenuhi standar #SuccessfulAging ; physical health, mental health, emotional state and social engagement. #LGBT #Aging #ageing #agingresearch #Jatim2KabehSedulur #JawaTimur #KhofifahEmilNomer1 DOI: 10.1016/j.jaging.2012.09.001

There's a rainbow in all of us! #FindARainbowDay #SuccessfulAging #OurFamilyTakingCareOfYourFamily

Yes, we like this comment and we like quinoa...yummy! #HealthyEating #successfulaging https://twitter.com/bestnutrition63/status/979825862236344321 …

#HealthyLife

Loyola-Chicago lifted by 98-year-old nun, fan in Sister Jean - #successfulaging #inspirationhttps://buff.ly/2DEMEBe

The Definition of Successful #Aging https://goo.gl/6PF31A  #successfulaging #aginggracefully @agingcarefl @AgingWisely @Aging_Society

How to Attain and Experience Successful Aging https://goo.gl/6PF31A  #successfulaging #aginggracefully @GIAging @healthyagingnet @changingaging

LIVE today at 10:32 am PST on KFI AM 640 YOUR BRAIN HEALTH MINUTE. #iheartradio The Official KFI AM #brain #health #coach #successfulaging #strategies #mentalfitness #healthylifestyle #exercise #meditation #nutrition #brainfoods #enhancedcognition #protectyourbrain

@Gail_Sheehy so excited to meet one of my favorite author at #SuccessfulAging

So, how many friends DO you need to age successfully? #friendship #successfulaging https://buff.ly/2FW9171

#SuccessfulAging: 7 Outta Sight Tips to Attain and Experience Its True Definition http://freemedsuppquotes.com/successful-aging-tips-definition/ …

Cathedral Village Receives Best of the Best Award from Masterpiece Living for Green Practices! #bestofbest #masterpieceliving #successfulaging https://buff.ly/2FOJGQt

Amazon ran out of books . . . again!! Yay!! A lot of us want to know more about #successfulaging #LoveYourAge

Cathedral Village Receives Best of the Best Award from Masterpiece Living for Green Practices! #bestofbest #masterpieceliving #successfulaging https://buff.ly/2FOJGQt

"Aging is a precious part of life and we need to do everything possible to engage with it, embrace it and use it as a positive force." #successfulaging https://buff.ly/2oCgtxu

#DFW: 9th in the nation in rapidly growing Aging Population, what are we doing to create a City for #SuccessfulAging? #SXSW #Dallas #Aging #CitiesAtSXSW @TheLBJSchool

What does it mean to age well? This important new book by Chris Stephens & Mary Breheny redefines what #successfulaging means. For more information: https://tinyurl.com/y7bnyra8  #criticalhealthpsych #agingresearch #aging

#Sanctuary4Longevity -- #HealthyAging: 'Our only real home is our bodies' "We definitely need new criteria and policies that recognize a social rather than medical model for #SuccessfulAging. #GlobalAgeing #GlobalHealth #AgeFriendly http://mailtribune.com/entertainmentlife/20180304/healthy-aging-our-only-real-home-is-our-bodies …

This Greenport resident has visited 81 countries — in retirement alone | Northforker https://buff.ly/2FrTkFj  #successfulaging #livingit!

The polls are open! Vote for the video that best represents Valuing Gray. #valuinggray #successfulaging https://buff.ly/2FJR5Nu

Thank you, Celebration Village Forsyth and Celebration Village Acworth, for sharing more about Masterpiece Living and Successful aging on your website. We're proud of our partnership with you. #successfulaging #MasterpieceLiving https://buff.ly/2orEhnM

Any change in the health care system that saves money and makes patients healthier deserves to be celebrated #SuccessfulAging @westhealth @ihihttps://www.nytimes.com/2018/02/25/opinion/hospitals-becoming-obsolete.html …

Challenge yourself at http://lookforthewoman.com  What is ageing successfully? http://lookforthewoman.com/ageing-successfully-or-successfully-ageing/ … #successfulaging #aging #ageism #disruptaging #frenchwomen #sexualharassment #lookforthewoman

Gadgets for #seniors: Ambitious techies roll out robots, smart gear for their #elders by @seungylee14 @mercnews via @chicagotribune http://www.chicagotribune.com/bluesky/technology/ct-biz-bsi-gadgets-for-seniors-20180207-story.html … #successfulaging #ageing #longevity

Our friends at @TheBirches share tips for starting a successful aging practice. Read more ... #successfulaging #MasterpieceLiving https://buff.ly/2EI8dWY

20 Journalists Chosen for Training on The Implications of an Aging Society & Working Longer http://nationalpress.org/newsfeed/20-journalists-chosen-for-training-on-working-longer/ … #Gerontology #EconomicsofAging #aginginplace #successfulaging

#Gerontology #successfulaging #caregiving —>https://twitter.com/agingparent/status/958478665439895553 …

Here's a poem @clrosenblatt wrote about the challenges of being a #dementia #caregiver: https://www.linkedin.com/pulse/dementia-caregiver-carolyn-rosenblatt-aging-expert-rn-attorney/?published=t …

Social connection is just as important for healthy aging as eating well and exercising. But it's easy to find yourself in a social slump. Check out these 25 ways to spice up your social life...#MasterpieceLiving #SuccessfulAging #HealthyAging http://ow.ly/ZMOf30irORP

20 Habits For a Healthier, Happier Life - https://buff.ly/2CSrXSK  #bluezones #successfulaging

LIVE at 12:43pm PST on KFI AM 640 YOUR BRAIN HEALTH MINUTE. The Official KFI AM 640 #brain #health #successfulaging #strategies #mentalfitness #healthylifestyle #nutrition #brainfoods #dietarytherapy #enhancedcognition #protectyourbrain @PinkConcussions @ConcussionMom @CogGevity

We've started a new blog series that will provide simple and practical tips for successful aging that you can apply in your day-to-day life. Check out the link below to learn more about #SuccessfulAging and what to expect from our new series... http://ow.ly/tQnN30iqoET

Expand your social network as you get older, and you’ll be on the path to #successfulaging.

Friendship Village of Bloomington was recently featured in the Mather LifeWays Institute on Aging’s annual Innovation at Work 2017 report as a Promising Practices Winner! Read more... #successfulaging #masterpieceliving https://buff.ly/2EcMdjv  @LifespaceComm

Some 80 million people will be older adults by 2050 and our national home-care infrastructure isn’t close to ready https://www.bloomberg.com/news/features/2018-02-09/americans-will-struggle-to-grow-old-at-home … via @BW #longtermcare #gerontology #successfulaging #economicsofaging

Research continues to validate that a successful aging lifestyle reduces hospital visits and depression, and increases overall healthy and longevity. Read more ... #successfulaging https://buff.ly/2DavInv

The Valuing Gray deadline has been extended through Friday, Feb. 16, 2018. Click here to submit your video! #valuinggray #successfulaging https://buff.ly/2GSxHhM

Taking into account a unique set of factors, such as safety, access to healthcare, financial security and mobility, @Investopedia named #DSMUSA a "Top 5 Best City Where Retirees Can Thrive." #successfulaging http://j.mp/2GRgl4T

The Mental Tricks of Athletic Endurance #wellness #fitness #weightloss #successfulaging #exercise #Athleticshttps://www.wsj.com/articles/the-mental-tricks-of-athletic-endurance-1517583851 …

Are you starting over after catastrophe? http://bit.ly/2nBEaVe  #successfulaging

What role will tech play to enable #SuccessfulAging? https://www.youtube.com/watch?v=ChgIvTbphmg … The human side of #DigitalHealth: Learn How http://Care.Coach  CareAvatarAn Animated Surveillance As Part of the #CareTeam to Support #Caregivers& #AgingParents #AgeInPlace https://www.wired.com/story/digital-puppy-seniors-nursing-homes/?mbid=social_twitter_onsiteshare …https://twitter.com/JonahComstock/status/943091439835209728 …

I love my work. But sometimes I wish I had time to dive deep on the human side of digital health, like this fantastic @WIRED piece: https://www.wired.com/story/digital-puppy-seniors-nursing-homes/ …

@jbwritetime Yes. #aging #successfulaging #gettingold #ageism #models #wellness #privilegehttps://twitter.com/thischairrocks/status/958036546426306560 …

Agreed! This is the underlying message of the "successful aging" model, which sets us up to fail and privileges the rich. https://twitter.com/jbwritetime/status/957908435361828864 …

#successfulaging #mindfulness #wellness #wellbeing #lifesatistaction #sociology #gerontology ~~~>https://twitter.com/emesfahanismith/status/905489505951592458 …

“Persuasive attack on the idea that happiness is the goal we should aim for... elegant...valuable.” — @WSJ. Order: http://ow.ly/YoyC30eXEqr

How can we change the perception of aging in America? Dr. Roger Landry shares his insights. #aging #ageims #successfulaging https://buff.ly/2ofCS5X

The Age Without Borders summit is in full swing. Be sure to tune in as more than 50 aging experts from around the world share their wisdom and experience. #caregiving #aging #successfulaging https://buff.ly/2E0SApO

Please take a few minutes to watch this special presentation I recently gave at Village on the Green. #successfulaging #humancapital #aging https://buff.ly/2mQyp5Y

Dr. Dave Comer and his team at Cambridge Village have incorporated lessons from both the MacArthur Foundation study on aging and the "Live Long, Die Short" philosophy. Read more... #successfulaging #MasterpieceLivinghttps://buff.ly/2CCFgXO

Click below to learn more about sponsorship opportunities for the 10th Annual Masterpiece Living Lyceum! #Lyceum18 #successfulaging https://buff.ly/2r8O2Lg

お腹がすいたら、よーしよーし！胃腸（消化活動）以外の細胞に、血液が回っている。機能が改善されている。と考える。#successfulaging #上手な年のとり方 #少食のすすめ

1日1個のりんごは医者を遠ざける。健康効果病気の改善効果が高まる。#successfulaging #上手な年のとり方 #少食のすすめ

@SunHealthAZ retirement communities, La Loma Village, The Colonnade and Grandview Terrace are Masterpiece Living partners that recognize the importance of social connection on health. #socialconnection #successfulaging https://buff.ly/2lWejHw

15 Simple Yet Effective Ab Exercises You Can Do at Home #wellness #successfulaging https://apple.news/APLRh5kMkQjGGGQxeKolxlw …

103-year-old dancer Eileen Kramer finds beauty in breath and joy in creativity. Learn more about this remarkable woman. #successfulaging #dance https://buff.ly/2p3IHnm
