# Supplementary material for: WordCommentsAnalyzer: A windows software tool for qualitative research
Source: F1000Res. 2018 Sep 4;7:536. Originally published 2018 May 3. [Version 2] doi: 10.12688/f1000research.14819.2 (PMC6137411; doi:10.12688/f1000research.14819.2)
Supplement: Peer review history

# Peer review: Referee response for version 2

**DOI:** 10.5256/f1000research.17614.r37967

**Published:** 2018-09-12

**Authors:** Yazdan Mansourian

**Affiliations:** 1 Charles Sturt University, Bathurst, NSW, Australia

I reviewed the new version. The amendments addressed my initial comments and the paper can be indexed now.

I have read this submission. I believe that I have an appropriate level of expertise to confirm that it is of an acceptable scientific standard.

# Peer review: Referee response for version 1

**DOI:** 10.5256/f1000research.16128.r36504

**Published:** 2018-08-02

**Authors:** Yazdan Mansourian

**Affiliations:** 1 Charles Sturt University, Bathurst, NSW, Australia

This article reports an overall description about developing a free and open source software designed for qualitative data analysis. This software assists researchers to organise and categorise the initial codes they create through data analysis process in various qualitative methods such as thematic analysis, ethnography and grounded theory. As this software is free and easily accessible, it will be useful for many researchers who do not have access to more sophisticated tools.

 The authors highlighted the ease of use and free access of this tool as its main benefits and I do agree with them. Nonetheless, they did not compare the capabilities and performance of this software with one or two well-known tools in this field to explain its strengths and weaknesses. Obviously, the existing tools have several options for deeper level of analysis and also some facilities for analysis of non-text data such as image, audio and video. As a result, I think the authors should also remind the reader about the inevitable limitations of this product. I also recommend the following revisions in the article:

 1. The article should be more informative in terms of distinctive features of this software comparing to similar tools in this context.

 2. The first citation in the introduction section (Lewis, 2004) has been published 14 years ago while since then the area of qualitative data analysis (QDA) has been developed considerably. As a result, I recommend to include a few recent citations in this section to provide the reader with a more accurate image of the current trends and issues in this area.

 3. The case presented in this article is based on a relatively small collection of data. Accordingly, we still do not know how effective the software will be in larger data sets. As sometimes, the huge volume of the data in large scale studies may reduce the performance of a software.

 4. The authors mentioned popular qualitative data analysis tools such as QDA Miner Lite does not support Persian and Arabic languages. Nonetheless, the case presented in the article is English and we still do not know about the real performance of this new tool in other languages such as Persian and Arabic.

 5. The conclusion is too brief at this stage and it should be more detailed and insightful. A good conclusion summaries the main points of the article and invites the reader to think further about the focal point presented in the discussions.

 In general, this article is based on a creative idea but still requires some revisions and I hope the software presented here will be useful for researchers who use qualitative approach in their studies.

I have read this submission. I believe that I have an appropriate level of expertise to confirm that it is of an acceptable scientific standard, however I have significant reservations, as outlined above.

# Response

**Published:** 2018-08-29

**Authors:** Ehsan Abdekhodaie

**Affiliations:** Iran

We thank Dr. Mansourian for his valuable comments about our manuscript/software. The comments surely helped us improve both the software and the manuscript.

 Some aspects of the software have been updated at the time we present this revised manuscript. Indeed, apart from the tweaks done in the software performance so that it can handle larger datasets, comparison of the software features to the features of other well-known QDA software compelled us to add some useful features to our software including search in code hierarchy feature and visualization tools. We also specifically respond to the reviewers comments in the following:

 1. We compared the features of this software to the similar tools in a new Table in the manuscript and added an accompanying paragraph which highlights the main points regarding this comparison and describes the specific limitations of our software. We also mentioned the limitations of WordCommentsAnalyzer regarding analysis of non-textual materials, doing complex queries, or presenting sophisticated visualizations.

 2. We added a few citations. Specifically, one to provide more recent data on the usage of popular QDA software (Woods, Paulus, Atkins and Macklin, 2016) and others to give the readers a glance on current challenges in learning complex QDA software (Silver and Rivers, 2014; Woods, Macklin, and Lewis, 2016).

 3, 4. The software indeed had issues with large dataset as the reviewer suspected. Consequently, we have updated the software so that it can handle larger datasets more efficiently. Then, we added another use case that is based on a collection of Iranian journal abstracts to test the software performance on a large dataset. This way we could also address the reviewer’s comment about performance of the tool in the presence of Persian/Arabic texts. It is worthy to note that, we developed the feature of search in the hierarchy and move in the hierarchy since we realized that it is not feasible for large datasets to find the codes by scrolling the hierarchy tree or to move the codes by merely dragging and dropping.

 5. We tried to make the conclusion more informative and more linked to the rationale of the study. Also, we revised it so as to present a better summary of the points made in the article.

# Peer review: Referee response for version 1

**DOI:** 10.5256/f1000research.16128.r34052

**Published:** 2018-06-11

**Authors:** Ronggui Huang

**Affiliations:** 1 Department of Sociology, Fudan University, Shanghai, China

Various QDA software provide similar functionalities in terms of coding operations and organization of codes, for instance, RQDA ( http://rqda.r-forge.r-project.org/), WeftQDA ( https://www.pressure.to/qda/), Py3QDA ( https://github.com/Ronggui/PyQDA/), among others.  A more systematic comparison of existing tools and *WordCommentsAnalyzer* will provide a clear picture on the relative advantages and disadvantages of the latter.

 It seems that *WordCommentsAnalyzer* mainly organizes the codes and shows the related coded text segments, but does not support coding operations on-the-fly. The operations of coding, remove coding, and re-coding have to be conducted on the Word Processor side. It would be helpful to potential users to describe this point clearly.

 Since the operation of coding must be done with Word Processor, it seems that coders have to remember and type the names of codes directly or via the copy-and-paste method. It would be valuable if coders can do the coding via *WordCommentsAnalyzer.*

 The most obvious advantage of *WordCommentsAnalyzer* is its easy use, especially for Windows users.

 Overall, *WordCommentsAnalyzer* is a valuable new tool for organizing codes based on Word Processor.

I have read this submission. I believe that I have an appropriate level of expertise to confirm that it is of an acceptable scientific standard.

# Response

**Published:** 2018-08-29

**Authors:** Ehsan Abdekhodaie

**Affiliations:** Iran

We would like to thank Dr. Huang for reviewing our software/manuscript and for his kind comments about the potential value this software provides. Here we respond to the reviewer‘s comments as follows:

 We added a ‘more systematic comparison’ of existing tools and WordCommentsAnalyzer through a table which included three commercial QDA tools and RQDA along with WordCommentsAnalyzer. We also discussed the relative pros and cons of the program.

 We asserted that our program is not meant to do coding operations on-the-fly and these operations should be done on the word-processing side.

 The reviewer mentioned the concern that it may be difficult for the researcher to memorize and type the names of codes or copy-and-paste them into Word. We added a feature that enables the user to drag and drop codes (from either the code list or the hierarchy tree) into the Word comment. So the user can both remain on the word-processing side for editing the files and reuse the developed code list/hierarchy effectively.
